# Community health workers recruitment from within: an inner-city neighborhood-driven framework

**DOI:** 10.1186/s13104-015-1700-0

**Published:** 2015-11-24

**Authors:** Hosseinali Shahidi, Cindy Sickora, Sharon Clancy, Roxanne Nagurka

**Affiliations:** Department of Emergency Medicine, Rutgers, New Jersey Medical School, 150 Bergen Street, Newark, NJ 07101 USA; Rutgers School of Nursing, 65 Bergen Street, Newark, NJ 07101 USA; Departments of Legal Management and Clinical Research, University Hospital, 150 Bergen Street, Newark, NJ 07101 USA

## Abstract

**Background:**

Community health workers (CHWs) are frontline public health workers who are trusted members of and/or have an unusually close understanding of the community served (APHA 2009). Among other roles, they are effective in closing critical communication gap between healthcare providers and patients as they possess key abilities to overcome cultural barriers, minimize disparities, and maximize adherence to clinical directions. In previous descriptions of the selection of CHWs, the role of community is clearly emphasized, but residence in the community is not indicated.

**Objective:**

We present an effective model of CHW selection by the community of members that reside in the community to be served.

**Methods:**

We outlined and implemented necessary steps for recruiting CHWs from within their target neighborhood between years 2011 and 2013. The identified community was an “isolated” part of Newark, New Jersey comprised of approximately 3000 people residing in three publicly-funded housing developments. We utilized a community empowerment model and established a structure of self-governance in the community of interest. In all phases of identification and selection of CHWs, the Community Advisory Board (CAB) played a leading role.

**Results:**

The process for the successful development of a CHW initiative in an urban setting begins with community/resident engagement and ends with employment of trained CHWs. The steps needed are: (1) community site identification; (2) resident engagement; (3) health needs assessment; (4) CHW identification and recruitment; and (5) training and employment of CHWs. Using an empowered community model, we successfully initiated CHW selection, training, and recruitment. Thirteen CHW candidates were selected and approved by the community. They entered a 10-week training program and ten CHWs completed the training. We employed these ten CHWs.

**Conclusions:**

These five steps emerged from a retrospective review of our CHW initiative. Residing in the community served has significant advantages and disadvantages. Community empowerment is critical in changing the health indices of marginalized communities.

## Background

The United States (US) spends more to finance its healthcare system than any country in the world [2]. Despite such per capita spending, US health indices (e.g., illness, infant mortality and life expectancy) continue to lag behind those of its peers [3]. The US is ranked last among eleven industrial countries on measures of health system quality, efficiency, access to care, equity, and healthy lives [4]. The current healthcare system ties revenue to the treatment of disease in lieu of its prevention [5]. Medical education is based on the model of “Find it and Fix it”. Promoting health and preventing illness requires significant time, effort, and investment. Attention to issues of cultural competency, increasing health literacy, and the ability to engage, empathize, educate and enlist patients are considered pillars of successful intervention by providers of healthcare [5]. The current medical care delivery system has time limitations, cultural barriers, and abundant gaps in communication [6, 7].

In recent years, Community Health Worker (CHW) have been introduced as new frontier workers who are either “from community or have significant familiarity with the community” to overcome some of these barriers by improving communication gaps, reducing cultural barriers, minimizing inequalities, increasing health literacy, promoting wellness, and maximizing adherence to medical directives [1]. Their ability to achieve these goals have been proven to be effective [2, 6, 7]. According to the recent Institute of Medicine Report, training of CHWs as part of a collaborative partnership between the community and a local healthcare delivery unit assures quality, supervision, and safety [2].

Empowering a community to take a leading role in improving the health and well-being of its members is critical; especially in communities that are marginalized, experience cultural incongruence with their healthcare providers, and/or mistrust the healthcare system [6–9]. The Centers for Disease Control (CDC), Community Preventative Services Task Force identified evidence gaps in CHW recruitment methods [10]. Many CHW recruitment efforts begin with healthcare providers initiating recruitment without involvement of the community being served [11]. In 1989, the World Health Organization stated “CHWs should be members of the communities where they work, should be selected by the communities, should be answerable to the communities for their activities, should be supported by the health system, but not necessarily a part of its organization, and have shorter training than professional workers.” [12] One way to provide a framework for empowerment is to request the community’s direct involvement. The implementation of such decisions are not well described or documented in the literature and attempts in giving communities the leading roles are not well described [8]. We provide a way that community empowerment plays the introductory role in identification and selection of CHWs, and describe successes and challenges to this process. This project review provides a five step framework for direct community involvement with the recruitment and subsequent training of CHWs from within that same community.

## Methods

### Community site identification and resident engagement

Newark is New Jersey’s largest city with a population of 277,000 people. It is a city riddled with crime, poor health and education outcomes, and a diverse population [13–17]. The community identified for this project is located in an “isolated” part of Newark, New Jersey and is comprised of approximately 3000 people residing in three public housing developments. The community was identified primarily for its characteristics: a primarily African-American community with a per capita income of approximately $11,000 per year; one of the poorest populations in the state [13]. The neighborhood is marginalized by geographic and environmental barriers. It is bordered by Port Newark one of the nation’s busiest ports, Newark International Airport, and one of the New Jersey Turnpike, one of the busiest traffic corridors in the country. It is also proximal to the Passaic River, an Environmental Protection Agency Superfund Clean-up site. Prior to our project, this community did not have the capacity or knowledge to increase their access to available resources or engage in activities that improve health and well-being. The community refers to itself as “forgotten”. Poor health literacy, scarce resources, and overwhelmed with burden of disease with minimal or for some no access to medical care.

The foundation for engaging the community in a self-governing structure and partnership with the local medical and nursing schools developed over several years by the lead faculty. Identifying key stakeholders and community leaders was a slow process and building a trusting relationship required commitment, continuous presence, and participation in community activities and gatherings. In 2007, a faculty member from the Rutgers School of Nursing discovered the community during a clinical rotation with community health nursing students. During the community assessment with students the demographics and lack of community resources were noted. In an effort to establish a relationship with the community, the students and faculty set-up weekly blood pressure screenings within the housing developments. The goal was to spend time and be visible to residents. Chalich and White contend that “hanging-out” may be one of the most important first steps in marketing a program in an underserved community and developing a relationship with the community [18]. The importance of relationship building and weekly presence established trust with residents and many confided confidential health information, supporting the need for a “health house.”

When funding for the project was secured from the Health Resources Services Administration (HRSA) of the US Department of Health and Human Services in 2010, a Community Advisory Board (CAB) was established and became the self-governing structure of the nurse managed health center or “health house” that would serve the three housing developments. The empowered community became an engaged partner in the new model of healthcare delivery at the site. Originally titled the Community Center for Health Empowerment and Care, the CAB renamed the center for two of its oldest residents both of whom had lived in the neighborhood for more than 50 years. The center became known as the Jordan and Harris Community Health Center (J&HCHC).

### CHW identification and recruitment process

**Fig. 1:**
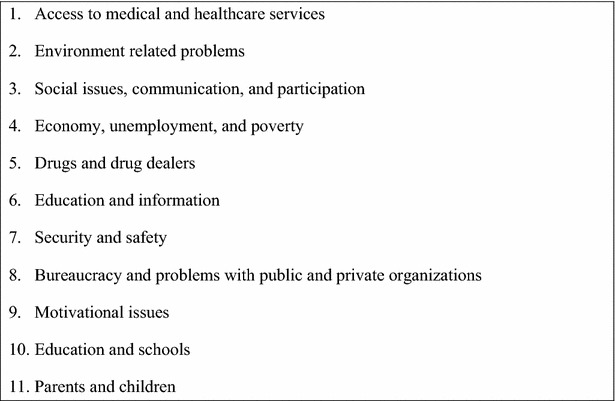
Community developed health need priority list; this figure lists the detailed health care needs of the community expressed by the community. We utilized this list to ensure the CHWs would be trained according to the needs of the community they would serve

A 2-day community needs assessment was conducted with many ordinary community members and the members of CAB. They participated in identifying the community needs and categorized them on the basis of priority and impact they believed would have on their health. The list was long and many of the identified problems were obvious, low health literacy, unhealthy life style, lack of preventive measures, environmental problems, lack of access to a reliable source of care with continuity and overwhelming burden of chronic disease interacting with complicated determinants of health (Fig. 1). The community-generated needs were essential in creating the training program for the CHWs.

Over several monthly meetings the historical background and the role CHWs have played in similar projects was described to the CAB. It was critical that the community have an understanding of the role in order for them to be actively engaged in the hiring process. The CAB provided an important community perspective on community need and how CHWs would fit into the healthcare delivery model that was evolving at the J&HCHC. They were very weary of the confidentiality issues that may arise from such encounters when the CHWs are from the community and reside in the community. The faculty from the medical and nursing schools committed to developing a curriculum that not only addressed CHW training, but also cultivated qualities that were important to the community. Grant funding was subsequently provided by the Healthcare Foundation of New Jersey (HCFNJ) for identification, training, and recruitment of the CHWs. The recruitment process started after HCFNJ funding was secured in year 2011. The CAB took the first step in recruitment by advising community members regarding the training and employment opportunity for residents.

Identification of CHWs was achieved with collaboration of the CAB. Community was informed of the opportunity to serve in a role that improves the health of the community. Recruitment criteria were developed in collaboration with the CAB and included the following: applicants had to be 18 years or older, reside in one of the three housing developments, have the ability to read and write in English and solve basic math problems. Those without previous health training were considered preferable as the faculty thought that nurse’s aides and medical technicians might bring a preconceived health perspective to the position that was not in line with the philosophy of the role that was developing for this project. CAB representatives had to approve each potential candidate for the role. After informing the community, the candidates were told to:

1. Complete and submit an application form
2. Write two short essays addressing the following:
  - What makes you believe you are a good candidate for the role of CHW?
  - Reasons you should be trained and hired.
3. Make an appointment for an interview with the healthcare team.

Eighteen candidates applied and participated in the interview. Each candidate was interviewed with two members of the team. A CAB representative also participated in the process. Each candidate’s essays were reviewed by the interviewers separately before the interview. The essays were reviewed to assess their ability to write, reason, and make inferences. During the interview, candidates were asked to explain why they wanted to join the healthcare team as a CHW and what they would bring to the job. The interviews provided the opportunity to evaluate whether the candidates understood the CHW role. Fifteen residents successfully completed the first round of the process with the interviewing team. The healthcare team brought these candidates forward for the next round.

**Fig. 2:**
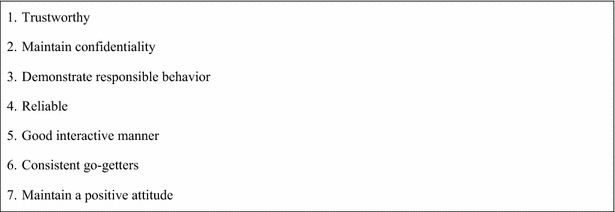
Community-generated list of essential CHW characteristics; this list represents the characteristics potential CHWs must possess in order to be approved by the CAB and accepted within the community they serve

The CAB identified a subcommittee with representatives from each of the three housing developments that would approve the final 13 CHW trainees. With the knowledge gained from the multiple discussions at monthly CAB meetings regarding the CHW role in other programs, the J&HCHC CAB subcommittee identified seven critical characteristics that potential candidates needed in order to meet the final qualifications for training and community need (Fig. 2).

The subcommittee identified those interviewees that would move forward for training by reviewing each candidate’s credentials and applied the seven criteria to see if they satisfy them. Two of the candidates did not meet all seven of the criteria and were disqualified by the CAB representatives mainly on the issue of trust (or lack of it) and the remaining thirteen were accepted into the training program.

## Results

CHW candidates were mostly women ranging in age from 20’s to 60’s and one male was the grandson of one of the other candidates. All were of African-American descent. Though we did not require a minimum education level, the majority of the trainees possessed a high school diploma or more. Being indigenous, they all reflected cultural attributes of the community they would ultimately serve. Seventy-five percent of our recruits completed their training and 90 % of those have remained employed after eighteen months. The thirteen candidate were enrolled in the training program and ten of them completed the required training and passed all the evaluations and formal tests. They were introduced to the community members in a graduation ceremony. They reflect the mosaic of the community they ultimately serve. Since the community was intimately involved in identification and selection of them, it was expected that they will be well received by their community members and would become agents of change. They had met the criteria set by the community and had satisfied the requirement. They were employed by the program to help in improving the health of their community members.

### Training and employment of CHWs

Our CHW training program was developed after an extensive review of existing curricula and training materials of similar urban projects including consultation with New Jersey’s Area Health Education Centers (NJAHEC) and the Community Health Worker Network of New York (CHWN-NY). Both offered critical information relative to the development of our training curriculum. We adapted and modified the NJAHEC curriculum which included health promotion and strategies for navigating the healthcare system. Our curriculum, a 10 week/80-h curriculum, was ultimately modeled after NJAHEC and CHWN-NY while using the previously described community’s needs as the foundation. Each module was arranged in 4-h instructions/once per week.

Two introductory modules have been previously tested by the CHWN-NY and provided an excellent launching platform for learning about the field of Community Health [19]. One session included detailed experiential exercises to learn about the stages of change model, a classic construct in the fields of health education and health behavior change).

The instructional sessions that followed the first two weeks of orientation were based on adult education models that minimize didactic presentations and emphasize role-playing, class exercises, case presentation, and interactive dialogue. At the end of each session, instructors reviewed the main points of the module and documented learner achievements via administration of a short quiz to measure knowledge gained. Two of the modules were used; one in the middle of the curriculum and one at the end for identifying the issues that were not clear and needed emphasis or reiteration.

During the course of the training, three candidates were dismissed due to absence from training and instructional sessions (attendance requirements had been established in advance with the candidates, the CAB, and the community-at-large). The remaining ten trainees successfully graduated from the program and were hired by our institution as per diem employees working 20 h per week, making $15.00/h.

## Discussion

CHW selection was a conscious well-informed decision by our collaborating community, the CAB, and healthcare team. People who live in the community have an intimate understanding of the strengths, weaknesses, barriers, and hardships faced by the community daily. The CHW hired for our program is a familiar face in the community, and is therefore able to easily navigate the cultural barriers and eliminate issues of mistrust historically inherent in medical and healthcare interventions. CHWs are able to reach out to friends and neighbors at all times and translate health promotion or health intervention messages in a way that is easily understood by community residents [20]. As members of the community, our CHWs know how to navigate the neighborhood day and night when non-indigenous healthcare providers may be hesitant related to the notion of community violence. Empowering communities to take a leading role in improving health and well-being of its members is critical [20]. One way to provide a framework for empowerment is to help the community members to realize their own potential and strength. Empowerment is a realization of such recognition and empowered people will be actively seeking opportunities to improve their life, including health and well-being.

In our model, the construct of community empowerment was established before introducing the idea of recruiting CHWs. A self-governing decision-making, community-elected, CAB had been previously established and was a mature functional group conducting regularly scheduled monthly meetings where health issues were discussed. The community itself developed this grassroots self-governing model. The empowered CAB directed the process by which CHWs were identified and recruited for subsequent training and employment. The healthcare team from nearby medical and nursing schools contributed to this process by providing information and guidance as needed.

We found most existing CHW programs have been designed to perform specific interventions previously decided by local healthcare providers, not the community itself [8]. Our model specifically asked the community to identify, categorize, and prioritize their needs. Health-related problems on their list were distinguished from those that were not health-related. Our healthcare team used a community generated list of health-related issues to construct the training curriculum for our CHWs.

This project demonstrated the fundamental value of community involvement with CHW programming. To ultimately improve health and well-being, specifically in marginalized urban communities, it is imperative that our healthcare system focus on functional methods to overcome the issues of disparities, cultural barriers, and communication. Lack of resources, barriers to healthcare access, and mistrust of “outsiders” suggests that we have to find new paradigms to address the well-being of marginalized communities. We presented a five step model for involving a CAB in recruitment, training, and employment of CHWs. It engages community insiders that were selected by the community, and employs these indigenous attaches within their own neighborhoods to help promote health, assist with oversight of medical directives, and navigate the complex healthcare system.

Over three years, the community health team of the school of nursing had gradually introduced itself to the community, participated in their daily activities, provided needed services and through building trust and partnership helped the community to organize itself and create a self- governing body with by-laws and regular monthly meetings (CAB). Through discussions during these meetings it became clear that the members of all three housing development are in dire need of life style changes and access to reliable sources of health and medical care. Specifically preventive and primary care. They had a genuine mistrust of the system. The burden of chronic diseases were overwhelming and medical care was piece meal and at best episodic.

Creating a system that would improves access, reduces inequalities, help modify the lifestyle, provide quality continuous primary care and is culturally acceptable and compatible with the community values and norms was planned. Jordan and Harris Community Health center was established through partnership with the community and school of nursing. It was funded by Health Services Resource administration and became the command center of health care intervention program.

To improve communication, cross the cultural barrier, assure compliance with medical directives and have a direct access to people in the community the role of CHWs was introduced into discussions. There was enough evidence that CHWs would bridge many existing gaps between providers of health care and people. The evidence was much more convincing where the communities were marginalized, culturally different and language barriers existed. CHW interventions had shown significant improvement in increasing health literacy, reducing inequalities, crossing the cultural and language barriers and making the suggestion of life style changes to reduce burden of chronic disease more acceptable.

Though the selection of CHWs from the community has been an integral part of our solution, it may have some disadvantages, such as patient confidentiality. We were initially concerned that confidentiality would be a major deterrent to sharing intimate life experiences with neighbors. As it turned out, the community stated “there are hardly any secrets in small contained communities.”

CHW supervision poses a major challenge and is a critical component of the project’s success or failure. The unique “unstructured” aspects of the program require self-direction and flexibility on the part of the CHW. However, this is a first time employment opportunity for many of our workers. Mentorship and supervision have proven to be critical to each worker’s success. Use of the internet, e-mail, completing daily reports, collecting data and communication with healthcare providers has required continuing education and close oversight. However, in spite of these limitations, CHWs know the best time and method to reach community members and this is the most important part of their role [20]. Cultivating creativity and allowing the CHWs to develop their own style empowers each to develop the role. It has taken us three years to develop a reporting system that provides flexibility and allows leadership the opportunity to oversee activities without developing barriers to CHWs’ success.

## Conclusion

To improve health and well-being, specifically in marginalized communities, it is imperative that a healthcare system focus on functional methods to overcome the issues of disparities, cultural barriers, and communication issues. Lack of resources, access to services, and mistrust of outsiders suggests that a different structure to address the well-being of these communities is needed. Our model engages insiders from the community, selected by the community, and employs these indigenous attaches within their home communities to help them promote health, translate and aid with medical directives and help them navigate the medial care system.

Future work will focus on quantitative results of selected indices of health and chronic disease management. Project sustainability remains a concern, as a viable means of supporting the CHW role has yet to be identified in the state of New Jersey. A method for reimbursement has not been established. This is a legislative issue making quantitative findings critical to the long-term viability of the work. Third-party payors are interested in the outcome data that is being generated as a result of this project. As we move forward we believe that the outcome data will support the role of CHWs in marginalized populations and that this model is replicable in a variety of community based settings.

## Abbreviations

CHW: Community health worker
US: United States
CAB: Community Advisory Board
NJAHEC: New Jersey’s Area Health Education Centers
CHWN-NY: Community Health Worker Network of New York

## References

[1] American Public Health Association. Policy number 20091: support for community health workers to increase health access and to reduce health inequities [online]. 2009. Available at: http://www.apha.org/policies-and-advocacy/public-health-policy-statements/policydatabase/2014/07/09/14/19/support-for-community-health-workers-to-increase-health-access-and-to-reduce-health-inequities.

[2] Committee on Quality of Health Care in America, Institute of Medicine (2001). Crossing the quality chasm: a new health system for the 21st century.

[3] Ginsburg JA, Doherty RB, Ralston JF, Senkeeto N, Public Policy Committee of the American College of Physicians (2008). Achieving a high-performance health care system with universal access: what the United States can learn from other countries. Ann Intern Med.

[4] Davis K, Stremikis K, Squires D, Schoen C. Mirror, Mirror on the wall: how the performance of the US Health Care System compares internationally. 2014 update, the commonwealth fund. 2014. Retrieved from http://www.commonwealthfund.org/publications/fund-reports/2014/jun/mirror-mirror.

[5] Keller VF, Carroll JG (1994). New model for physician-patient communication. Patient Educ Couns.

[6] Krieger J, Collier C, Song L (1999). Linking community-based blood pressure measurement to clinical care: a randomized controlled trial of outreach and tracking by community health workers. Am J Public Health.

[7] Birkel RC, Golaszewski T, Koman JJ (1993). Findings from the horizontes acquired immune deficiency syndrome education project; the impact of indigenous outreach workers as change agents for injection drug uses. Health Educ Q.

[8] O’Brien MJ, Squires AP, Bixby RA (2009). Role development of community health workers: an examination of selection and training processes in the intervention literature. Am J Prev Med.

[9] Wallerstein N, Duran B (2010). Community-based participatory research contributions to intervention research: the intersection of science and practice to improve health equity. Am J Public Health.

[10] CDC. A handbook for enhancing CHW programs: recruitment. Retrieved from http://www.cdc.gov/cancer/nbccedp/pdf/trainpdfs/hb-recruitment.pdf.

[11] Reninger BM, Barroso CS, Mitchell-Bennett L (2010). A. Process evaluation and participatory methods in obesity-prevention media campaign for Mexican Americans. Health Promot Pract..

[12] WHO. Strengthening the performance of community health workers in primary health care. Report of a WHO Study Group. Geneva, World Health Organization (WHO Technical Report Series, No. 780); 1989. 2497585

[13] U.S. Census Bureau: state and county quickfacts. Data derived from population estimates, American community survey, census of population and housing, county business patterns, economic census, survey of business owners, building permits, census of governments. Retrieved from http://www.quickfacts.census.gov/qfd/states/34/3451000.html.

[14] Newark NJ. Real estate and demographic information, http://www.neighborhoodscout.com/nj/newark/.

[15] State of New Jersey, Department of Health. New Jersey State Health Assessment, http://www.4state.nj.us/dhss-shad/query/selection/birth/BirthSelection.html.

[16] Data Resource Center for Child and Adolescent Health, http://www.childhealthdata.org/browse/survey/results?q=219&r=32.

[17] Goldman D, McGlynn E (2001). US health care: facts about cost, access, and quality.

[18] Chalich T, White JP (1997). Providing primary care to poor urban women. Nurs Forum.

[19] Community Health Worker Network of New York City. Retrieved from http://www.chwnetwork.org/.

[20] Ingram M, Sabo S, Rothers J (2008). Community health workers and community advocacy: addressing health disparities. J Community Health.

